# Reversible aripiprazole-induced tardive dyskinesia: a case series from Saudi Arabia and literature review

**DOI:** 10.3389/fpsyt.2026.1749247

**Published:** 2026-02-18

**Authors:** Sultan M. Alshahrani, Lujain Mohammed Althagafi, Jameelah Abdullah Saeedi, Lina Mohammed R. Aljohani, Aqeel A. Almarhoon, Ahmed S. Alyahya

**Affiliations:** Department of Neurosciences, King Abdullah bin Abdulaziz University Hospital, Princess Nourah bint Abdulrahman University, Riyadh, Saudi Arabia

**Keywords:** aripiprazole-induced tardive dyskinesia, aripiprazole, aripiprazole-related tardive dyskinesia, case series, reversible tardive dyskinesia, Saudi Arabia, tardive dyskinesia

## Abstract

Tardive dyskinesia (TD) is a slow-onset, hyperkinetic movement disorder involving repetitive involuntary movements. It is classified as a medication-induced movement disorder that occurs in association with the use of dopamine-blocking agents–most commonly, antipsychotics. It is classically understood as an unremitting process, with a few cases of reversible conditions and uncertain remission rates. Aripiprazole possesses a distinct and unique mechanism that decreases its risk compared to other antipsychotics. In this case series, we discuss three cases involving Saudi women who developed aripiprazole-induced TD; notably, all cases remitted upon aripiprazole discontinuation or dose reduction without utilizing other forms of treatment. The current literature remains limited in describing aripiprazole-induced TD, lacking a clear understanding of the development of TD following aripiprazole administration, the determined remission rate, and definite management.

## 1 Introduction

Tardive dyskinesia (TD) is a slow-onset, hyperkinetic movement disorder that involves repetitive, involuntary movements ranging from slight tremors to uncontrollable choreatic movements of the whole body. It presents mainly with orofacial movements, namely oral–buccal–lingual involuntary movements. However, in approximately half of all patients, it also involves the peripheral extremities and may involve the neck or trunk in 25% of patients. In severe cases, it can affect swallowing, speech, and mastication. In rare cases, respiratory muscle involvement can present and be life-threatening (1, 2). TD is classified as a medication-induced movement disorder that occurs in association with the use of dopamine-blocking agents, most commonly antipsychotics, either during exposure or shortly after withdrawing from the offending agent (four weeks in cases of oral medications and eight weeks in cases of long-acting intramuscular injections) (1). Initially, physicians were disinclined to accept TD as a drug-related disorder owing to its similar phenomenology to several neurological disorders and its odd clinical course; it does not strictly appear with drug induction or dose increase, and it does not remit upon discontinuing the offending agent. Nevertheless, it is currently recognized as an iatrogenic drug-related disorder (3).

The underlying pathophysiology of TD remains uncertain; however, the most accepted theory links TD to the dopamine receptor hypersensitivity hypothesis. Prolonged blockade of dopamine D2 receptors by antipsychotics or other blocking agents prompts a compensatory upregulation and an increase in receptor density. Consequently, this adaptation leads to an exaggerated postsynaptic reaction to dopamine (1–4). Nevertheless, this does not sufficiently explain why TD may persist for many years when the offending agent is discontinued (5). This hypothesis has been supported by the examination of the vacuous chewing movement (VCM) in animal models. In these studies, an exaggerated response to dopamine agonists was observed following the introduction of a single dose of dopamine-blocking agents. However, the exaggerated response subsided after discontinuation of dopamine-blocking agents, indicating a reversible phenomenon (6). This does not correlate with the clinical picture of TD, with the usual late onset, individual vulnerability, appearance of symptoms without utilizing dopamine agonists, and potential irreversibility even after stopping the offending agent. In addition, these results have not been replicated in human studies (4–6). Moreover, different theories have been reviewed with mixed outcomes, several inconsistencies, and insufficient proof of the roles of serotonin receptors (specifically 5-HTR2A), oxidative stress, γ-glutamyl transferase (GABA), glutamate-related excitotoxicity, vesicular monoamine transporter 2 (VMAT2), and maladaptive synaptic plasticity (4, 7).

Several risk factors have been recognized including female sex, advanced age, long-term exposure to dopamine-blocking agents, mood disorders, negative symptoms in schizophrenia, intellectual disability, diabetes mellitus, and prior extrapyramidal symptoms (8). Nonetheless, TD can be recognized in familial patterns, suggesting a genetic link. Genetic testing of *DRD2*, *VMAT2*, and HTR2A polymorphic variations has been strongly suggested to be linked to TD. Additionally, variations of hepatic enzymes involved in drug metabolism, like cytochrome P450, have been linked to the risk of TD as well. Specifically, *CYP2D6* (encoding a protein involved in the metabolism of aripiprazole and several other antipsychotics) is highly polymorphic with more than 100 allelic variations producing different rates of metabolism and enzyme activity. TD has been linked to variations that yield either slower or more rapid metabolism rates (8–10).

The annual incidence of TD is estimated to be 2–5%, and its prevalence is estimated to be 25.3% (2, 11). Although, it is mainly associated with dopamine-blocking agents, several case reports have described the use of anticholinergic agents, antidepressants, antiemetics, anticonvulsants, antihistamines, antiparkinsonism agents, and stimulants (2). Antipsychotics are further classified into first- and second-generation antipsychotics (FGAs and SGAs, respectively), where the latter have a lower risk of extrapyramidal side effects, including TD, owing to their lower affinity to the D2 receptor within the dorsal striatum and their associated 5-HT2A/2C serotonin receptor antagonism (2). In two large meta-analyses, FGAs were estimated to have an annual incidence rate of developing TD of 6.5% (95% confidence interval [CI]: 5.3–7.8%) and a prevalence of 30% (95% CI: 26.4–33.8%). Comparatively, SGAs were estimated to have an annual incidence rate of 2.6% (95% CI: 2.0–3.1%) and a prevalence of 20.7% (95% CI: 16.6–25.4%) (11, 12).

Aripiprazole, similar to SGAs, is associated with a lower risk of developing TD than FGAs. Furthermore, aripiprazole has the lowest risk of developing TD compared to SGAs when compared to all FGAs (13). This is likely due to the action of aripiprazole as a partial agonist of the D2 receptor rather than a pure D2 antagonist; it acts as a dopamine system stabilizer by acting as a functional antagonist in areas of dopamine hyperactivity and as a functional agonist in areas of dopamine hypoactivity. Thus, it is suggested that it be classified as a third-generation antipsychotic (2, 7, 14, 15), which can help mitigate and even resolve TD caused by other antipsychotics. Although large-scale studies are required, many reports consistent with this finding have been published (7, 15, 16). Nevertheless, aripiprazole can still lead to the development of TD through uncertain processes as well, with an estimated prevalence ranging from 0.2% to 3.4% (7).

In this study, we investigated three cases of TD related to the use of aripiprazole. Notably, all patients achieved remission after discontinuation of aripiprazole.

## 2 Methodology

This case series was conducted at the Department of Neurosciences, King Abdullah bin Abdulaziz University Hospital (KAAUH) in Riyadh, Saudi Arabia. Ethical approval was obtained from the Institutional Review Board (IRB), and written informed consent was obtained from all participants.

A literature search was conducted using PubMed/MEDLINE to identify published cases of aripiprazole-induced TD. The search was conducted using “Aripiprazole AND Tardive Dyskinesia” and “Aripiprazole AND Tardive syndrome” and included articles published up to October 2025. We included all cases with aripiprazole being associated with TD. We excluded cases describing tardive syndromes other than TD, such as tardive dystonia. We also excluded cases presenting with pre-existing movement conditions.

## 3 Case description

### 3.1 Case 1

A 57-year-old female patient diagnosed with bipolar affective disorder (BAD) was maintained on aripiprazole (15 mg/day) and quetiapine (300 mg at bedtime). After approximately four and a half years of stability on these regimens, the patient exhibited involuntary movements of the tongue and lip, with no other extrapyramidal symptoms. Prior to this regimen, she took olanzapine that was stopped a few months later due to over-sedation. No other antipsychotics were utilized. Her family history was unremarkable for TD. Her medical history was positive for hypothyroidism controlled with thyroxine (50 mcg daily). She did not smoke or consume alcohol. Aside from in-and-out tongue protrusion and lip smacking that she could not control, no other focal neurological deficits were detected, physical examination was otherwise unremarkable, and vital signs were within normal limits. The Abnormal Involuntary Movement Scale (AIMS) score was calculated to be 15 for the involuntary movements. Accordingly, the dosage of aripiprazole was gradually decreased until cessation, whereas quetiapine was maintained on the same dose of 300 mg daily. Shortly after aripiprazole discontinuation, tongue movements became partially subject to voluntary control (she could shortly inhibit them while attempting to do so). In the subsequent 2 months, her dyskinetic movements progressively diminished and ultimately resolved upon cessation. At the 1-year follow-up, the patient remained asymptomatic.

### 3.2 Case 2

A 62-year-old female patient, diagnosed with BAD and aerophobia (fear of flying), was treated with aripiprazole at a dose of 5 mg/day for obsessive ruminative thoughts concerning her daughter, which was then titrated to 15 mg/day. The concomitant drugs comprised quetiapine (200 mg) at bedtime, bupropion 300 mg daily, and lamotrigine 200 mg daily. Shortly after, she showed akathisia that was managed sufficiently with propranolol 10 mg three times per day. However, 6 months after starting aripiprazole, she exhibited involuntary facial and lip movements and hand tremors that did not respond to propranolol. While she had not been exposed to other antipsychotics while receiving care at our clinics that was initiated about 3 years before initiating aripiprazole, previous exposure to other antipsychotics could not be established and was limited by an inability to conduct her earlier medical records and by the patient’s inability to recall. Although her family history was positive for BAD and major depressive disorder, no family member had experienced symptoms suggesting TD. Her medical history was positive for hypertension managed with lercanidipine (10 mg daily) and dyslipidemia managed with rosuvastatin (20 mg daily). She did not smoke or consume alcohol. Aside from this involuntary movement, no focal neurological deficit was detected, the physical examination was normal, and vital signs were within normal limits. The AIMS score was 14 for the involuntary movement. Aripiprazole was promptly terminated, propranolol was discontinued, and the quetiapine dosage was elevated to 300 mg/day, while other medications were maintained at the same doses. The patient showed gradual improvement after discontinuation of aripiprazole, with total symptom resolution at the 1-month follow-up and no recurrence throughout the 1-year follow-up while being treated with the last regimen.

### 3.3 Case 3

A 26-year-old female patient diagnosed with schizoaffective disorder was maintained on aripiprazole 10 mg/day for approximately 1 year, achieving clinical stability. Subsequently, she received a single intramuscular long-acting injection of aripiprazole (400 mg), after which she developed involuntary jaw clenching, neck twisting, and irregular, purposeless left arm movement. She did not have any previous exposure to other antipsychotics, and her medical history was unremarkable. There was no family history of such movement or other movement disorders. She did not smoke or consume alcohol. Her physical examination was otherwise unremarkable, and vital signs were within normal limits. Laboratory tests were unremarkable and within normal ranges. The AIMS score was calculated to be 18 for the involuntary movement. The patient discontinued aripiprazole treatment without any additional pharmacological intervention. Over the next 2 months, her dyskinetic symptoms gradually improved, with complete remission thereafter. Oral aripiprazole was resumed without symptom recurrence. The patient remained clinically stable and symptom-free during the 1-year follow-up period.

## 4 Discussion

This case series presents three cases of aripiprazole-induced TD. All patients were administered aripiprazole, either orally or via depot injection, and the symptoms resolved completely with discontinuation without requiring any other intervention. Although TD has historically been considered an unremitting pathology, reports involving remitted (i.e., reversible) cases have been published (17–29). Larger studies examining possible reversibility are still needed to provide a realistic understanding, estimation of outcomes, and possible factors that may contribute to condition improvement or resolution (3). One retrospective cohort study of 108 subjects followed up for 3 years following withdrawal of the offending agents revealed the development of tardive syndromes, including TD and multiple other clinical disorders. In this analytical study, tardive syndrome resolved in 13% of the subset, and of those, only 2.8% improved with discontinuation of the offending agent (30).

Management of TD remains a challenge and the available strategies remain suboptimal. The general approach to TD is broadly similar among different antipsychotics and include a revision of the offending agent and evaluating the clinical feasibility of dose reduction and discontinuation. Switching to another antipsychotic with a relatively lower TD risk, such as quetiapine or clozapine, should be considered, especially if TD has developed following treatment with an FGA. For persistent, distressing symptoms, adding a VMAT2 inhibitor (valbenazine or deutetrabenazine) should also be considered. Other management strategies still require stronger evidence (31–33).

Regarding the reversibility of aripiprazole-induced TD, in addition to the lack of large-scale studies, published cases have shown mixed outcomes, adding further to the debate on the possible reversibility and remission of TD. Many published case reports have indicated an irreversible course (22, 23, 34–38). Several case reports have been presented where TD resolved by stopping aripiprazole while also requiring another intervention (**Tables 1**, **2**) (17–26), and three case reports have reported resolution of TD with only the discontinuation of aripiprazole. This finding is consistent with the results of our case series (**Table 3**) (27–29).

**Table 1:** Cases of aripiprazole-induced TD improved by stopping aripiprazole with utilizing another low TD–risk SGA.

Case report/year	Age/ sex	Underlying disorder	Prior antipsychotics	Aripiprazole dose	Treatment duration	TD onset	Management	Outcome
Evcimen et al./2007 (17)	54/Female	Schizoaffective disorder	Prior use of olanzapine for 5 years	20 mg/day	10 months	After 10 months	Aripiprazole discontinued, maintained on quetiapine	Dyskinesia resolved completely within 1 month
Abbasian and Power/ 2009 (18)	24/Female	Paranoid schizophrenia	2 weeks on risperidone	15 mg/day	9 months	After 9 months of treatment	Aripiprazole cross-tapered with quetiapine 200 mg BD	Symptoms remitted completely within 4 weeks
Tomruk et al./ 2011 (19)	56/Female	Schizoaffective disorder	Haloperidol, risperidone, amisulpride, chlorpromazine	30 mg/day	1 year	After 1 year on aripiprazole alone	Aripiprazole was discontinued and quetiapine was initiated	Rapid improvement
Ono et al./ 2012 (20)	22/Male	Schizophrenia	Prior use of olanzapine	24 mg/day	6 months	Started with olanzapine, improved with aripiprazole 12 mg and recurred with 24 mg	Aripiprazole was discontinued and started quetiapine (50 mg/day)	Dyskinesia improved completely within weeks
She et al./ 2018 (21)	29/Female	Schizophrenia	Neuroleptic-naive other than aripiprazole	10 mg/day	8 years	After 8 years of treatment	Aripiprazole stopped; quetiapine (200 mg/day) and Ginkgo biloba (240 mg/day) administered	Effectively relieved within 3 months
She et al./ 2018 (21)	29/Female	Schizophrenia	Neuroleptic-naive other than aripiprazole	10 mg/day	10 years	After 10 years of treatment	Aripiprazole stopped; quetiapine (200 mg/day) and Ginkgo biloba (240 mg/day)	Effectively relieved within 3 months
Godhania et al./ 2024 (22)	23/Male	BAD	Ongoing use of aripiprazole in the community with mild TD	200 mg IM depot	Not specified	Marked increase in dyskinesia after starting depot injection	Aripiprazole switched to quetiapine 400 mg BD	AIMS reduced from 20 to 2 after several weeks

TD, tardive dyskinesia; SGA, second-generation antipsychotic; BAD, bipolar affective disorder; IM, intramuscular injection; BD, twice per day; AIMS, Abnormal Involuntary Movement Scale.

**Table 2:** Cases of aripiprazole-induced TD improved by stopping aripiprazole and utilizing other interventions.

Case report/year	Age/ sex	Underlying disorder	Prior antipsychotics	Aripiprazole dose	Treatment duration	TD onset	Management	Outcome
Wang et al./ 2009 (23)	52/Female	Schizophrenia	Prior use of sulpiride for 6 years	10 mg/day	2 months	After 2 months of aripiprazole only	Diphenhydramine 150 mg/day	Gradually disappeared in 3–4 months
Lungu et al./ 2009 (24)	62/Male	PTSD	Neuroleptic-naive other than aripiprazole	15 mg/day	18 months	After 18 months of treatment	Aripiprazole stopped; symptoms persisted and was unresponsive to trihexyphenidyl, baclofen, clonazepam, and botulinum toxin. Bilateral GPi-DBS was done.	DBS led to considerable benefit within 3 months
Alexander & Bickerstaff/ 2013 (25)	46/Female	BAD, borderline traits	History of substance abuse; previous risperidone-induced TD, resolved over 2 weeks of stopping	10 mg/day	2 weeks	Reemergence of TD after 2 weeks on aripiprazole	Aripiprazole discontinued, clonazepam 0.5 mg	“Good effect” timeframe not mentioned
Aguilar et al./ 2019 (26)	23/Female	Psychosis	Prior use of olanzapine	Up to 25 mg/day	6 years	After 6 years of treatment	Aripiprazole discontinued. combination of tetrabenazine (75 mg/day), botulinum toxin, and clozapine (100 mg/day)	Significant improvement, timeframe not mentioned

TD, tardive dyskinesia; PTSD, post-traumatic stress disorder; BAD, bipolar affective disorder; GPi, globus pallidus internus; DBS, deep brain stimulation.

**Table 3:** Cases of aripiprazole-induced TD improved with dose reduction or discontinuation only.

Case report/year	Age/ sex	Underlying disorder	Prior antipsychotics	Aripiprazole dose	Treatment duration	TD onset	Management	Outcome
Maytal et al./ 2006 (27)	62/Female	MDD, GAD, agoraphobia	Ziprasidone and risperidone	15 mg/day	18 months	After 18 months of treatment	Aripiprazole discontinued	Symptoms resolved completely within 2 weeks
Caykoylu et al./ 2010 (28)	45/Female	Unspecified psychotic disorder	Prior use of quetiapine (200 mg/day) for 3 years	Up to 15 mg/day	3 months	After 3 months of treatment	Dose reduced to 10 mg/day, added biperiden 3 mg/day and TD improved, but worsened after stopping biperiden. Aripiprazole was stopped and TD resolved.	Movements resolved within 1 week
Patra/ 2016 (29)	19/Male	Paranoid illness	Neuroleptic-naive other than aripiprazole	20 mg/day	6 months	After 6 months, upon dose increase of fluoxetine	Dose reduced to 15 mg/day	Movements were reduced after 2 weeks

TD, tardive dyskinesia; MDD, major depressive disorder; GAD, generalized anxiety disorder; BAD, bipolar affective disorder; IM, intramuscular injection; LAI, long-acting injection.

In conclusion, TD is classically recognized as a chronic and unremitting disorder (2). This may be partly explained by the fact that most of the patients switched to and were maintained on other antipsychotics instead of the causative agents owing to clinical necessity, underestimating the true remission rates (30). However, studies show that aripiprazole-induced TD can be improved and resolved, which is consistent with our findings (3, 7, 14, 15, 17–30). These observations may highlight the possibility of reversing TD if promptly recognized and managed, possibly with stopping the offending agent only (13). In short, the available data remain limited. Controlled trials, systematic reviews, and statistical studies are warranted to comprehend the prognostic factors associated with reversibility, estimate the true remission rate, and provide appropriate management (3). Moreover, we strongly encourage research that compares different antipsychotics in terms of TD remission rates to guide prescribing physicians (2, 3). Finally, careful monitoring and early detection are essential to optimize outcomes (5, 13).

## Data Availability

The raw data supporting the conclusions of this article will be made available by the authors, without undue reservation.
